# Metabolic Signature of Articular Cartilage Following Mechanical Injury: An Integrated Transcriptomics and Metabolomics Analysis

**DOI:** 10.3389/fmolb.2020.592905

**Published:** 2020-12-17

**Authors:** Jennifer Southan, Emily McHugh, Heather Walker, Heba M. Ismail

**Affiliations:** ^1^Department of Infection, Immunity and Cardiovascular Disease, Medical School, The University of Sheffield, Sheffield, United Kingdom; ^2^biOMICS Mass Spectrometry Facility, Department of Animal and Plant Sciences, The University of Sheffield, Sheffield, United Kingdom

**Keywords:** cartilage, injury, osteoarthritis, transcriptomics analysis, metabolomics, arginine metabolic pathways, glycolysis (glycolytic pathway)

## Abstract

Mechanical injury to the articular cartilage is a key risk factor in joint damage and predisposition to osteoarthritis. Integrative multi-omics approaches provide a valuable tool to understand tissue behavior in response to mechanical injury insult and help to identify key pathways linking injury to tissue damage. Global or untargeted metabolomics provides a comprehensive characterization of the metabolite content of biological samples. In this study, we aimed to identify the metabolic signature of cartilage tissue post injury. We employed an integrative analysis of transcriptomics and global metabolomics of murine epiphyseal hip cartilage before and after injury. Transcriptomics analysis showed a significant enrichment of gene sets involved in regulation of metabolic processes including carbon metabolism, biosynthesis of amino acids, and steroid biosynthesis. Integrative analysis of enriched genes with putatively identified metabolite features post injury showed a significant enrichment for carbohydrate metabolism (glycolysis, galactose, and glycosylate metabolism and pentose phosphate pathway) and amino acid metabolism (arginine biosynthesis and tyrosine, glycine, serine, threonine, and arginine and proline metabolism). We then performed a cross analysis of global metabolomics profiles of murine and porcine *ex vivo* cartilage injury models. The top commonly modulated metabolic pathways post injury included arginine and proline metabolism, arginine biosynthesis, glycolysis/gluconeogenesis, and vitamin B6 metabolic pathways. These results highlight the significant modulation of metabolic responses following mechanical injury to articular cartilage. Further investigation of these pathways would provide new insights into the role of the early metabolic state of articular cartilage post injury in promoting tissue damage and its link to disease progression of osteoarthritis.

## Introduction

Cartilage is a unique connective tissue with only one cell type – the chondrocytes – embedded in a rough matrix rich in collagen II fibers and proteoglycan contents. Cartilage is an aneural and avascular tissue (Huber et al., 2000), making it a model connective tissue to study the direct responses arising from cells in response to mechanical injury. The low surface attrition and high lubrication of articular cartilage allow the smooth surface articulation of opposing joint surfaces and the protection of cartilage surface from shear stress (Seror et al., 2015). Cartilage collagen fibers are oriented and highly cross-linked, which aids the efficient joint articulation and provides the matrix with high tensile properties (Palmoski et al., 1979; Vanwanseele et al., 2002). Articular cartilage chondrocytes are highly mechanosensitive cells although they rarely divide in healthy cartilage tissue and may appear quiescent (Soder et al., 2002; Tesche and Miosge, 2004; Kvist et al., 2008).

Mechanical injury is a cause of articular cartilage damage and is the main risk factor in the development of osteoarthritis; a degenerative disease affecting the synovial joints which is characterised by deterioration of the articular cartilage (reviewed in Lohmander et al., 2007; Thomas et al., 2017). Although OA is a common and disabling disease currently affecting at least 250 million worldwide (Prieto-Alhambra et al., 2014), its pathogenesis is poorly understood. There is still no disease-modifying medical therapy for OA, which creates a major socioeconomic burden in the population (Hunter and Bierma-Zeinstra, 2019).

Recently, a number of studies have employed large-scale omics approaches to understand OA as a heterogeneous disease with a purpose to phenotype and stratify patient groups and to identify early disease biomarkers. Metabolomics profiling provides an unbiased comprehensive analysis of metabolites as the end products of cellular processes in a biological system. Several studies employed either targeted or untargeted metabolomics analysis using NMR or LC-MS platforms in different tissues from OA patients’ synovial joints including the bone, cartilage, and synovial tissues while other studies focused on the analysis of body fluids from OA patients as synovial fluid, serum, and urine (Lamers et al., 2005; Shet et al., 2012; Mickiewicz et al., 2015; Jin et al., 2016; Marchev et al., 2017; Carlson et al., 2018; Hinata et al., 2018). These studies revealed possible common metabolic changes associated with OA including amino acid metabolism, glycolysis, TCA cycle, and ATP biosynthesis (reviewed in Rockel and Kapoor, 2018).

The cartilage damage in OA may be caused by maladaptive responses to repetitive mechanical injury. Using murine and porcine *ex vivo* models of cartilage injury, we previously showed that mechanical injury to cartilage rapidly activates the intracellular signaling pathways characteristic of the inflammatory response including TAK1 (Ismail et al., 2017), NFκB, MAP kinases (Vincent et al., 2002; Gruber et al., 2004; Watt et al., 2013; Ismail et al., 2017), and Src (Watt et al., 2013) followed by induction of genes characteristic of inflammation (Chong et al., 2013; Ismail et al., 2017). Using these models of early cartilage injury is a useful tool to identify the cellular responses of articular cartilage to mechanical injury insults. This will provide a deep understanding of the key pathways modulated by injury and give insights into how early responses to injury would induce tissue damage leading to pathological injury-linked diseases as seen in OA.

Here we have identified the metabolic signature of articular cartilage post mechanical injury using an integrative analysis of transcriptomics and global metabolomics profiling of murine femoral epiphyseal hip cartilage before and after injury. Furthermore, we performed a global metabolomics profiling of another cartilage injury model using porcine metacarpophalangeal joints. A summary of the study design and analysis pipeline is shown in Supplementary Figure 1. Integrative gene–metabolite analysis of murine cartilage injury and the cross-species analysis showed a significant enrichment of genes and putative metabolites involved in amino acids and carbohydrate metabolism, in particular, arginine biosynthesis and related metabolic pathways, glycolysis/gluconeogenesis, and vitamin B6 metabolic pathways. These results show that early cellular responses to mechanical injury involve a rapid modulation of metabolic pathways that could provide a metabolic phenotype and early markers for cartilage tissue response to injury. Further investigation is required to understand the role of the early modulation of these metabolic pathways in promoting the tissue damage seen in OA.

## Materials and Methods

### *Ex vivo* Murine and Porcine Cartilage Injury Models

Injury to the murine hip cartilage was induced by avulsion of femoral epiphysis of freshly culled 5–6-week-old mice as described previously (Chong et al., 2013). Pig trotters (front feet) of freshly slaughtered 3–6−month−old pigs were purchased from a local farm and used as a source of cartilage. Injury to porcine articular cartilage was induced by explantation from the articular surface of the metacarpophalangeal (MCP) joints of the pig trotters as previously described (Watt et al., 2013). Cartilage was then snap-frozen (0 h) or cultured for indicated times post injury in serum-free medium. Following incubation, cartilage was snap-frozen and stored at −80°C until further processing.

### RNA Extraction and Microarray Analysis

#### RNA Extraction

Six murine cartilage hips were combined from three freshly culled animals for each time point as one biological replicate to provide sufficient RNA amount. Three independent biological replicates were prepared for two samples sets (control and injured cartilage). Hips from each replicate were homogenized in 1 ml TRIzol (Invitrogen) for 1 min on ice; then, the homogenate was left at room temperature for 5 min and mixed with 200 μl of 1-bromo-3-chloropropane (BCP). Samples were vortexed for 15 s then incubated at RT for 5 min followed by centrifugation at 13,000 rpm for 15 min at 4°C. The aqueous layer was separated and used for further purification using a Zymo-Spin RNA cleanup kit (R1013, Zymo Research) as per manufacturer’s instructions. RNA quality was tested by RNA Bioanalyzer before proceeding with microarray analysis.

#### Microarray Data Analysis

RNA was prepared and hybridized to Illumina mouse BeadChip microarrays following manufacturers’ protocols. Raw data were imported into R statistical software (https://www.r-project.org/) for further processing and analysis using Bioconductor packages. Raw signal intensities were background-corrected (using array-specific measures of background intensity based on negative control probes) and then transformed and normalized using the “vsn” package (Gentleman et al., 2004; R Core Team, 2007). Further statistical analysis and visualization of the results were performed using Perseus software (Tyanova et al., 2016). Microarray data have been submitted to the Gene Expression Omnibus (accession number: GSE155892). Pathway analysis of enriched gene sets was performed using STRING (https://www.string-db.org/) (Szklarczyk et al., 2019) and WebGestalt databases (http://www.webgestalt.org/) (Liao et al., 2019).

### Metabolite Extraction and Global Metabolomics Analysis

The same processing procedure was followed for extraction of metabolites from porcine and murine cartilage samples. Samples were prepared in triplicates for each time point. Each replicate from murine samples included six hips while a replicate from porcine samples is for the cartilage surface explanted from a single trotter MCP joint. Cartilage samples were mixed with pre-chilled solvent mixture containing methanol/chloroform/water (2.5:1:1, by volume) to 100 mg tissue dry weight. Tissue was ground using a FastPrep-24 5G homogenizer (MP Biomedicals). Samples were vortexed, kept on ice for 5 min, and then centrifuged at 14,000 rpm for 2 min at 4°C. Supernatant was then removed into a pre-chilled tube. Pellet was re-extracted with pre-chilled solvent mixture containing methanol/chloroform (1:1 by volume) then vortexed and incubated on ice for 10 min. New extracts were centrifuged, and supernatants were collected then combined with supernatants of the first extraction step. Combined supernatants were mixed with pre-chilled ddH2O and chloroform then centrifuged at 14,000 rpm for 15 min at 4°C. Cleared extracts were separated into two phases (an aqueous phase and an organic phase), and the aqueous phase was retained and stored at −80°C prior to analysis.

Metabolite extract aqueous phases were analyzed in positive and negative ion modes using a Waters Acquity UPLC coupled to a Waters SYNAPT G2-Si time-of-flight (ToF) mass spectrometer with electrospray sample introduction (ESI) (Waters Corporation, United States). The instrument was calibrated prior to analysis using sodium formate over the mass range 50–1,200 amu. Samples were introduced directly into the mass spectrometer using the UPLC as an automated injector and using leucine enkephalin as a post-processing lock mass standard in order to obtain highly accurate mass measurements. Each sample was run in triplicate, and the spectra from these technical replicates were combined to select only those peaks present in all three technical replicates. Data were collected over the mass range 50–1,200 *m*/*z* with a scan time of 1 s per scan. Data were then processed using an in-house Excel macro based on Overy et al. (2005) for noise reduction and binning of data. The macro also assigned putative IDs by matching accurate *m*/*z* peaks to the BioCyc.org database – the encyclopedia of genes and metabolism (https://humancyc.org/) using a tolerance of 20 ppm. IDs are putatively identified by accurate mass and manually cross-checked against the Human Metabolome Database (HMDB) (https://hmdb.ca/) and mummichog (http://mummichog-2.appspot.com/) (Li et al., 2013) database. A more detailed protocol for cartilage metabolite extraction and metabolomics analysis is available upon request.

### Statistical and Pathway Analyses

Metabolite mass-to-charge ratios (*m*/*z*) and their corresponding peak average intensity tables were imported into Perseus software 1.6.12.0 (Tyanova et al., 2016). Data were filtered based on valid values, and only metabolites that are successfully detected in the three biological replicates of each time point were used for further analysis. Data were then log-transformed and values missing from normal distribution were imputed from the analysis using default parameters of imputation function in Perseus. Differential enrichment of metabolite features between injured and control groups was analyzed using two-tailed Student’s *t*-tests FDR-adjusted *p*-value less than 0.05. Enriched metabolite features were mapped to relevant pathways using mummichog (Li et al., 2013) and MetaboAnalyst 4.0 (Chong et al., 2019). Lists of enriched genes and metabolites post injury of murine hips were used in the joint pathway module for integrative analysis in MetaboAnalyst 4.0. Pathways reported were significant by pathway overrepresentation analysis with an FDR-adjusted *p*-value less than 0.05.

## Results

### Transcriptomics of Articular Cartilage Post Injury Shows a Significant Enrichment of Gene Sets Involved in Regulation of Metabolic Processes

To identify the specific gene sets modulated by articular cartilage injury, we performed a microarray transcriptomic analysis of hip murine cartilage before and after injury. Injury to cartilage was induced by avulsion of the femoral epiphysis as described previously (Chong et al., 2013) and either snap-frozen (CTRL) or cultured for 4 h post injury. Samples were then prepared and analyzed as described in the “Materials and Methods.”

**FIGURE 1:**
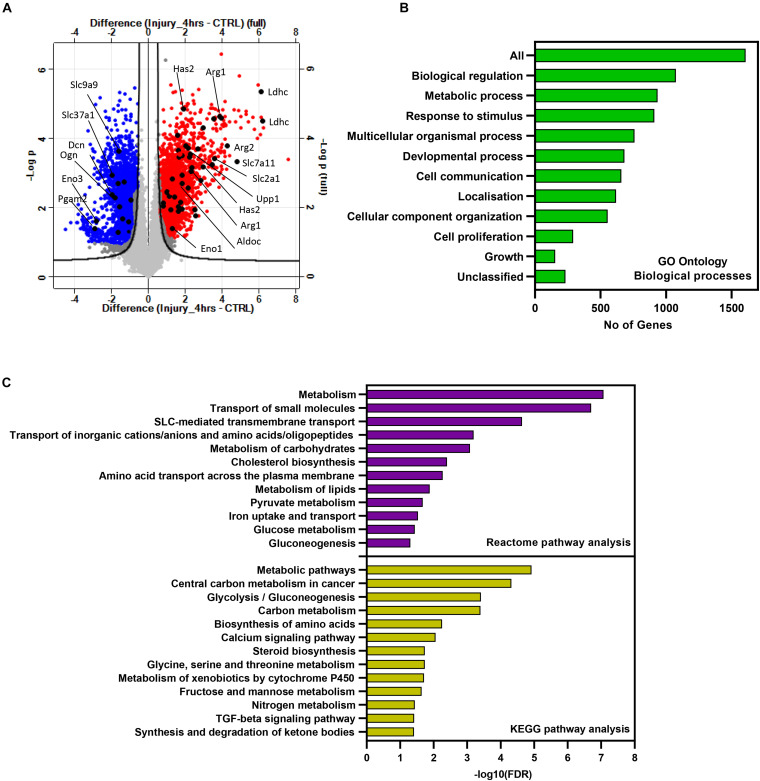
Injury to murine cartilage significantly modulates the expression profile of a large set of genes involved in regulation of metabolic processes. Microarray transcriptomic analysis of murine cartilage hips before and after injury was performed as described in “Materials and Methods.” **(A)** Volcano plot of gene expression profiles in injured cartilage compared to control. Differentially regulated genes post injury are highlighted in red (upregulated) and blue (downregulated). Examples of modulated metabolic genes are labeled in black. Genes significantly regulated in response to injury were analyzed for pathway enrichment using WebGestalt and STRING databases. **(B)** Gene Ontology biological processes of enriched genes post injury. Analysis shows that more than 800 genes are involved in metabolic processes. **(C)** Enriched metabolic pathways post injury retrieved from the STRING database using the list of significantly modulated genes.

Microarray data were then filtered and analyzed using Perseus software version 1.6.12.0 (Tyanova et al., 2016). Differentially enriched genes post injury with a fold of change >2 and FDR-adjusted *p*-value less than 0.05 were considered significant. Injury to murine hip cartilage resulted in a significant modulation of a large set of genes. A group of 769 genes was significantly downregulated while a group of 946 genes was differentially upregulated in hip cartilage chondrocytes post injury. Figure 1A shows a volcano plot of differentially regulated genes in the injured set compared to control. Pathway analysis was then performed on differentially enriched genes using WebGestalt and STRING databases. The bar chart in Figure 1B shows enriched Gene Ontology (GO) biological processes. Interestingly, more than 800 genes were involved in metabolic processes. Using the STRING database, a set of 621 enriched genes was mapped to “regulation of metabolic processes” (GO:0019222). KEGG pathway analysis showed a significant enrichment of genes involved in glycolysis, amino acid biosynthesis, and fructose and mannose pathways. Reactome analysis showed enrichment of 200 genes involved in metabolism including carbohydrate metabolism and amino acid transporters (Figure 1C). Examples of significantly modulated metabolic genes post injury are labeled on the volcano plot in Figure 1A including genes encoding for lactate dehydrogenase (Ldhc), arginase 1 (Arg1), arginase 2 (Arg2), enolase 3 (Eno3), aldolase c (Aldoc), and glucose transporter 1 (Slc2a1). Details of enriched genes in metabolic pathways are shown in Supplementary Table 1.

The strong enrichment of genes post injury linked to metabolic processes raised the possibility that injury to articular cartilage would change the metabolic state of chondrocytes and consequently the tissue metabolic profile. For that, we extended the analysis and performed global metabolomics profiling of murine hip cartilage before and after induction of tissue injury as described below.

### Mechanical Injury to Murine Hip Cartilage Significantly Modulates the Tissue Metabolic Profile

Metabolomics profiling provides deep and comprehensive insights into the tissue state in response to a stimulus. To identify the metabolic signature of cartilage in response to mechanical injury, we performed untargeted metabolomics profiling of murine hip cartilage before and after injury. Hip cartilage was pooled from three freshly culled animals per time point, and cartilage was either snap-frozen (CTRL) or cultured at 1 hour (triplicates) and 2 hours (duplicates) post injury. Metabolite extracts were prepared and then analyzed in negative and positive ion modes using mass spectrometry as described in the “Materials and Methods.”

**FIGURE 2:**
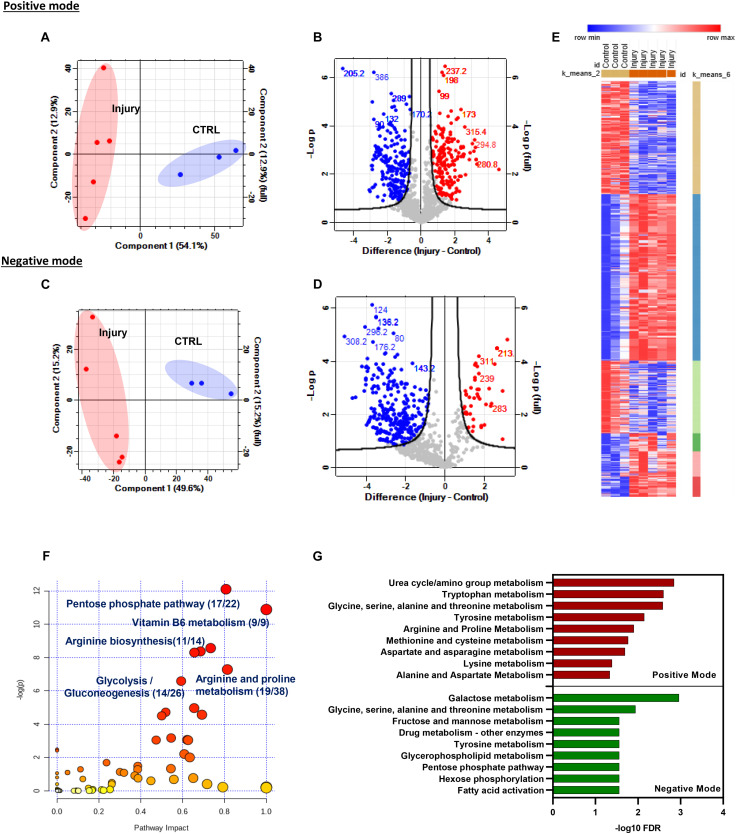
Mechanical injury to murine hip cartilage significantly modulates the tissue metabolic profile. Injury to murine hips was induced, and tissue metabolites were extracted followed by global metabolomics profiling of the tissue before and after injury using LC–MS. Extracted metabolites were analyzed in positive and negative ion modes as described in “Materials and Methods.” Principal component analysis and volcano plots of detected metabolites in positive mode **(A)** and negative mode **(B)**. Enriched metabolite features post injury are labeled in red (upregulated) and blue (downregulated). Numbers on volcano plots represent spectral bins of enriched features post injury in positive mode **(C)** and negative mode **(D)**. **(E)** *K*-mean clustering of enriched-metabolite post hip cartilage injury shows six clusters of modulated features that could be distinguished based on their profile post injury. **(F)** Top enriched metabolic pathways post cartilage injury were identified using the modulated metabolite features list in the MetaboAnalyst 4.0 pathway analysis module. Numbers shown are of matched putative metabolite hits per total number of metabolites in each pathway. **(G)** Top enriched pathways of differentially modulated *m*/*z* metabolites features in positive and negative ion modes using mummichog metabolomics analysis software (http://mummichog-2.appspot.com/).

Peak intensities from control and injured samples were analyzed using Perseus software. A large number of *m*/*z* features were detected in each mode. A total of 4,160 *m*/*z* features were detected in positive mode, and 1,890 *m*/*z* features were detected in negative mode in all samples. Differentially modulated metabolite features were detected by comparison of combined injury samples with uninjured controls. Principal component analysis (PCA) showed a strong separation between samples based on phenotype and distinguished injured sets from uninjured sets in both modes (Figures 2A,C).

We have observed that injury caused a differential regulation of putative metabolite levels in both modes. A total of 414 accurate peaks mapped to 202 spectral bins were differentially modulated in negative mode while 760 unique accurate peaks were differentially modulated in positive mode and mapped to 266 spectral bins (volcano plots in Figures 2B,D and Supplementary Table 2). *K*-mean clustering analysis of differentially regulated metabolite features post injury in both modes identified six different clusters of metabolite sets (Figure 2E).

Accurate peak lists of enriched features retrieved from both modes were annotated as described in “Materials and Methods” and analyzed for pathway enrichment using MetaboAnalyst 4.0 (Figure 2F) and mummichog databases (Figure 2G). Interestingly, amino acid and carbohydrate metabolic pathways are significantly enriched using both databases. Examples of the top enriched metabolic pathways post injury are metabolism of vitamin B6, arginine biosynthesis, glycolysis/gluconeogenesis, and arginine and proline metabolism. Details of enriched pathways and matched putative metabolites are described in Supplementary Table 3.

Taken together, mechanical injury to cartilage significantly changed the metabolic state of the tissue as seen by significant modulation of several putative metabolic features. Top modulated features were mapped to amino acid and carbohydrate metabolism with significant involvement of arginine and proline metabolism.

### Integrative Analysis of Transcriptomics and Metabolomics Profiles of Murine Articular Cartilage Post Injury

**FIGURE 3:**
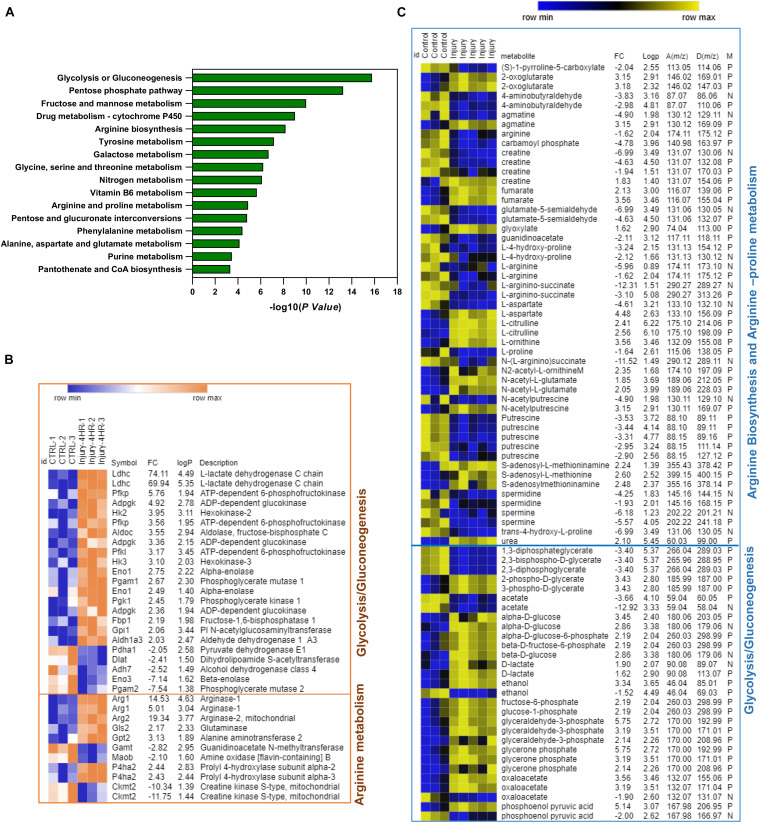
Integrative pathway analysis of differentially regulated genes and putative metabolite features post injury shows significant enrichment of arginine biosynthesis, arginine/proline metabolism, and glycolysis/gluconeogenesis metabolic pathways. **(A)** Top enriched pathways using genes–metabolites joint pathway analysis module in MetaboAnalyst 4.0. **(B)** Heat map of significant genes post injury in arginine metabolism and glycolysis/gluconeogenesis. **(C)** Heat map of putative metabolite features enriched in arginine metabolism and glycolysis/gluconeogenesis. D(*m*/*z*) for detected peaks, A(*m*/*z*) for accurate peaks, M for mode, N for negative ion mode, and P for positive ion mode. In **(B,C)** Fc is for gene expression or putative metabolite feature fold of change in injured cartilage compared to uninjured control. LogP is for –log10 of *p*-value identified by Student’s *t*-test. Heat maps were generated using MORPHEUS matrix analysis software (https://software.broadinstitute.org/morpheus/).

To identify the joint gene–metabolite pathways, we performed an integrative analysis of enriched genes and metabolite features retrieved from the transcriptomic microarray analysis and metabolomics profiling of hip cartilage. The lists of enriched genes and metabolites with folds of changes were used for joint pathway analysis in MetaboAnalyst 4.0 as described in the “Materials and Methods.” The organism was specified as *Mus musculus*. The bar chart in Figure 3A shows the top significantly enriched metabolic pathways with integrated genes. A significant enrichment was observed for carbohydrate metabolism as glycolysis, galactose, and glycosylate metabolism as well as TCA cycle and amino acid metabolism (valine, leucine, cysteine, methionine, and arginine biosynthesis). The heat maps in Figures 3B,C show genes and putative metabolite features modulated in response to injury in arginine biosynthesis, arginine–proline metabolism, and glycolysis/gluconeogenesis metabolic pathways (integrated gene–metabolite map in Supplementary Figures 2, 3). Details of genes and metabolites enriched in all pathways are described in Supplementary Table 4.

In arginine biosynthesis, Arg1, glutamic pyruvate transaminase (Gpt2), and glutaminase (Gls) genes were significantly upregulated post injury together along with L-ornithine, urea, and *N*-acetyl glutamate putative metabolite features, while four metabolite features were significantly downregulated including carbamoyl phosphate, L-citrulline, L-arginine, and *N*-(L-arginino)succinate. An enrichment of four genes (Gamt, P4ha1, Ckb, and Moab) and 12 putative metabolite features linked to arginine and proline metabolism was also observed (Figures 3B,C). In glycolysis and gluconeogenesis pathways, Ldhc, Aldoc, Pgam1, Gpi, Aldh1a3, HK2 and HK3, Adpegk, and Pfk1 genes were significantly upregulated while Pgam2, Dlat, Adh7, Pdha1, and Eno3 genes were downregulated. The levels of putative metabolite features matching lactate, glycerone phosphate, oxaloacetate, glucose, glyceraldehyde-3-phosphate, phosphoenol pyruvate, and fructose-6-phosphate were upregulated while features of glycerol phosphate, 2,3-bisphospho-D-glycerate, and acetate were significantly downregulated (Figures 3B,C and Supplementary Figure 4).

This shows that mechanical injury to articular cartilage modulates gene expression and metabolite features linked to arginine biosynthesis, arginine–proline metabolism, and glycolysis and gluconeogenesis.

### Metabolomics Profiling of Porcine Cartilage Post Injury

To validate our findings in another *ex vivo* cartilage injury model, we used porcine metacarpophalangeal joints. Cartilage was explanted from these joints as described in the “Materials and Methods.” Two time series were analyzed in cartilage post injury: a short time series (0, 5, 10, and 30 min) and a longer time series at 1, 2, 4, and 24 h post injury in triplicates.

**FIGURE 4:**
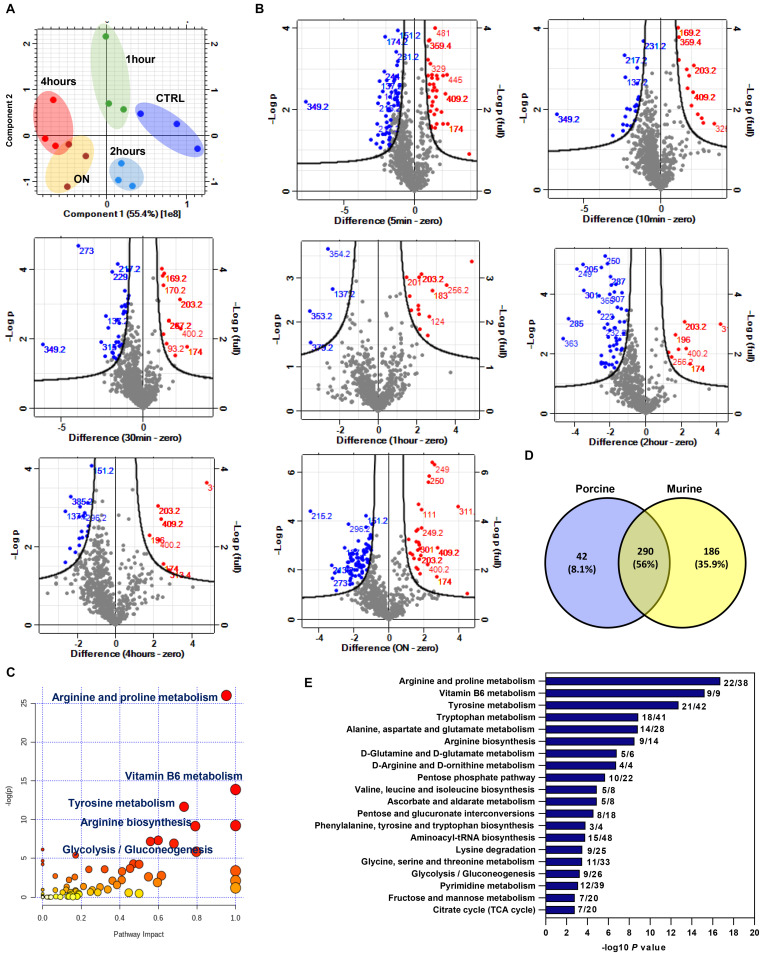
Enriched metabolite features and metabolic pathways post mechanical injury in porcine articular cartilage and cross analysis with differentially modulated features in injured murine cartilage. **(A)** Principal component analysis (PCA) of metabolite features in porcine cartilage tissue samples after 0, 1, 2, and 4 h and overnight (ON) post injury (combined positive and negative ion modes). **(B)** Volcano plots of detected metabolite features post cartilage injury at indicated times compared to uninjured control. Numbers on plots are for annotated enriched metabolite features spectral bins. Upregulated features after injury are shown in red, and downregulated features are shown in blue compared to control. **(C)** Pathway analysis of enriched metabolite features post injury to porcine cartilage using MetaboAnalyst 4.0. **(D)** Cross analysis of enriched features (*m*/*z*) in murine and porcine cartilage post mechanical injury. **(E)** Pathways commonly enriched in murine and porcine cartilage. A list of 290 common metabolite features between the two *ex vivo* cartilage injury models was used for pathway analysis in MetaboAnalyst 4.0. Numbers on the bar chart are for the matched metabolite hits per total number of metabolites in each pathway.

Metabolites were extracted and analyzed as described in the “Materials and Methods.” Features detected in both modes were combined for further analysis in Perseus software. For clarity, PCA is shown for the longer (hours) time series only (Figure 4A), which showed a good separation of samples based on injury induction time. Injury resulted in a rapid modulation of several metabolite features as early as 5 min. Volcano plots in Figure 4B show the spectral bins of enriched metabolite features post injury in both modes at indicated time points. A set of 48 putative metabolite features were commonly enriched in all time points post injury. A unique set of metabolites was enriched at 24 h (Supplementary Figure 5). Retrieved matched features/compound hits were used for pathway enrichment analysis in MetaboAnalyst 4.0. We have observed a significant enrichment of 20 metabolic pathways (Figure 4C and Supplementary Table 5) including arginine and proline metabolism, arginine biosynthesis, vitamin B6 metabolism, and carbohydrate metabolic pathways.

These results showed that a rapid response of porcine articular cartilage chondrocytes is observed following mechanical injury. Pathways involved in amino acid and carbohydrate metabolism are at the top of the enriched-pathways list, implicating the importance of these pathways in early response to injury in the cartilage tissue.

### Cross Analysis of Porcine and Murine Cartilage Untargeted Metabolomics Post Injury

To identify commonly enriched metabolic pathways post injury in porcine and murine cartilage injury models, enriched putative metabolite features identified in both analyses were cross analyzed with Venny 2.0.1 (Figure 4D). A set of common 290 putative metabolite features were commonly enriched post injury. Interestingly, this set showed a significant enrichment for arginine biosynthesis pathway and arginine and proline metabolism. Other pathways involved in amino acids and carbohydrate metabolism are also significantly enriched as shown in Figure 4E. A complete list of enriched pathways and matched putative metabolites is listed in Supplementary Table 6.

## Discussion

Mechanical injury is a cause of articular cartilage damage and is linked to OA. The articular chondrocytes are highly mechanosensitive cells which respond very rapidly and efficiently to an injury insult. Understanding the cellular response of chondrocytes to mechanical injury will give insights into how injury would induce tissue damage and lead to pathological injury-linked diseases as seen in OA.

To understand how mechanical injury modulates the chondrocyte transcriptome and metabolome and how these levels of cellular regulation are integrated, we performed transcriptomics and metabolomics analyses of murine cartilage before and after injury. We extended the analysis further to include an *ex vivo* porcine cartilage injury model to compare and validate the metabolomics data obtained from the murine injury model. The transcriptomics analysis of murine cartilage showed a significant enrichment of a large number of genes post injury. Interestingly, almost half of the enriched genes are either related to or involved in the regulation of metabolic processes and metabolic pathways such as amino acid synthesis and transport, as well as carbohydrate metabolism (Figure 1 and Supplementary Table 1).

Integrative analysis of transcriptomics and metabolomics of murine hip cartilage post injury showed modulation of genes and metabolites linked to a number of amino acids and carbohydrate metabolic pathways (Figure 3 and Supplementary Table 4). We observed a significant enrichment of arginine biosynthesis and related proline metabolic pathways. A significant gene expression induction of Arg1, Arg2, Gpt2, and Gls genes was observed accompanied by increased levels of putative metabolite features of ornithine and urea as products of L-arginine degradation, which conforms to significantly reduced features of arginine post injury. Levels of arginine feature were also significantly reduced post injury in all time points in porcine cartilage. We also observed increased levels of L-citrulline and fumarate while levels of L-arginino-succinate and carbamoyl phosphate features were significantly reduced post injury (Figure 3 and Supplementary Figure 2). A number of metabolic pathways use arginine as a substrate in a competitive way to produce either L-citrulline/nitric oxide through a reaction activated by nitric oxide synthase (NOS) (Abramson et al., 2001) or urea and L-ornithine through arginase, which is encoded by the Arg1 gene. Arginase is a manganese-containing enzyme essential for the disposal of toxic ammonia by converting L-arginine to L-ornithine and urea in the final step of the urea cycle (Caldwell et al., 2018). Arginase contributes to collagen synthesis and factors involved in fibrosis through further metabolism (Wehling-Henricks et al., 2010). Interestingly, in OA patients, ornithine is increased while the ratio of arginine to ornithine is decreased compared to that in controls (Zhang et al., 2016). Arginine is significantly depleted in plasma of refractory knee OA patients (Zhang et al., 2016). Our results also showed induced gene expression of Gpt2, which is accompanied by increased levels of 2-oxaloglutarate and decreased levels of alanine metabolite features. The Gpt2 gene encodes alanine aminotransferase enzyme, which is involved in L-alanine degradation through activation of the reversible transamination reaction between 2-oxaloglutarate and alanine to form glutamate and pyruvate (Yang et al., 2002). These data highlight the importance of arginine metabolism/biosynthesis in cartilage hemostasis and its response to mechanical injury.

A set of enriched putative metabolite features post injury was linked to proline metabolism. For example, proline and hydroxyl proline in both murine and porcine cartilage models were significantly reduced post injury. We have found four genes enriched in our integrative analysis known to be involved in arginine/proline metabolism including P4ha1, which was upregulated while Ckm, Gamt, and Moab genes were downregulated post injury. Gamt and Ckm genes are involved in the conversion of guanidinoacetate to phosphocreatine (Da Silva et al., 2009). Intermediate metabolite features in this reaction were also downregulated post injury compared to control. Other metabolite features linked to arginine and proline metabolism were significantly reduced following injury in both injury models including spermine, spermidine, creatine, glutamate-5 semialdehyde, agmatine, 4-aminobutyraldehyde and putrescine while S-adenosyl methionine and S-adenosyl methioninamine were significantly upregulated (Supplementary Figure 3). These results reflect a possible suppression of arginine–proline metabolism following mechanical injury to the articular cartilage, which may lead to inhibition of protein synthesis as proline metabolism is essential for this process (Wu et al., 2011).

Interestingly, in both *ex vivo* cartilage injury models, we have observed a modulation of amino acid features (Supplementary Tables 3, 5) including valine and tryptophan, arginine, proline, glycine, alanine, glutamine, leucine, and isoleucine. Functional amino acids are beneficial and would play an anti-inflammatory role in healthy tissues. OA development was linked to alterations in amino acid metabolism including glutamate and arginine family amino acids as well as their related metabolites (e.g., creatinine, hydroxyproline, γ-aminobutyrate, dimethylarginine, and homoarginine) (reviewed in Li et al., 2016).

We have also observed a significant enrichment of genes and metabolite features linked to glycolysis/gluconeogenesis following injury including Ldhc, Pfkp, Adpgk, HK2, Aldoc, HK3, Eno1, Pgk1, Gpi1, and Aldh1a3 genes accompanied by upregulation of metabolite features of glucose, lactate, oxaloacetate, phosphoenol pyruvate, glycerine phosphate, glucose-6-phosphate, and phosphoglycerate (Figure 3 and Supplementary Figure 4). This implicates upregulation of glycolysis as a source of energy production, which is needed for cartilage repair post injury stress. Another set of genes and metabolites linked to glycolysis/gluconeogenesis was downregulated post injury, highlighting a possible two-tier mechanism involved in this pathway regulation. Interestingly, synovial fluids from OA patients showed higher levels of metabolites involved in both glycolysis and TCA cycle compared to rheumatoid arthritis (RA) patients (Anderson et al., 2018) while TCA intermediates malate, citrate, succinate, and fumarate showed higher levels in late stages of OA compared to early stages of the disease (Kim et al., 2017).

Overall, our findings suggest that mechanical injury to the articular cartilage modulates a set of genes and metabolites involved in arginine biosynthesis and related metabolism as well as glycolysis/gluconeogenesis pathways. Data suggest an enhanced arginine metabolism upon injury accompanied by reduced proline metabolism, which may occur to suppress/halt protein synthesis. Injury stress response is also accompanied by a two-tier regulation of glycolysis needed for energy production with several genes integrated (upregulated and downregulated). This initial response may provide a chance for the cartilage tissue to repair itself and provide the essential energy needed to perform these reparative reactions. The study provides some basic clues on the responsive nature of the local environment of articular cartilage to mechanical injury on gene and global metabolic levels. This early metabolic signature of injury may reflect cellular events promoting tissue damage in OA when the tissue fails to repair itself. Further targeted analysis of the putative metabolite features identified in this study is essential in cartilage injury models and in comparison to diseased cartilage samples to specify the direct role of injury-enriched metabolic pathways in tissue damage.

## Data Availability Statement

The datasets presented in this study can be found in supplementary material and online repositories. The names of the repository/repositories and accession number(s) can be found in the article.

## Author Contributions

HI developed the concept, designed the study and experiments, and wrote the manuscript. HI, JS, EM, and HW performed the experiments and data analysis. HW revised the submitted version. All authors contributed to the article and approved the submitted version.

## Conflict of Interest

The authors declare that the research was conducted in the absence of any commercial or financial relationships that could be construed as a potential conflict of interest.
