# Supplementary material for: Metabolic Signature of Articular Cartilage Following Mechanical Injury: An Integrated Transcriptomics and Metabolomics Analysis
Source: Front Mol Biosci. 2020 Dec 17;7:592905. doi: 10.3389/fmolb.2020.592905 (PMC7773849; doi:10.3389/fmolb.2020.592905)
Supplement: Supplementary file 1 [file Data_Sheet_1.pdf]

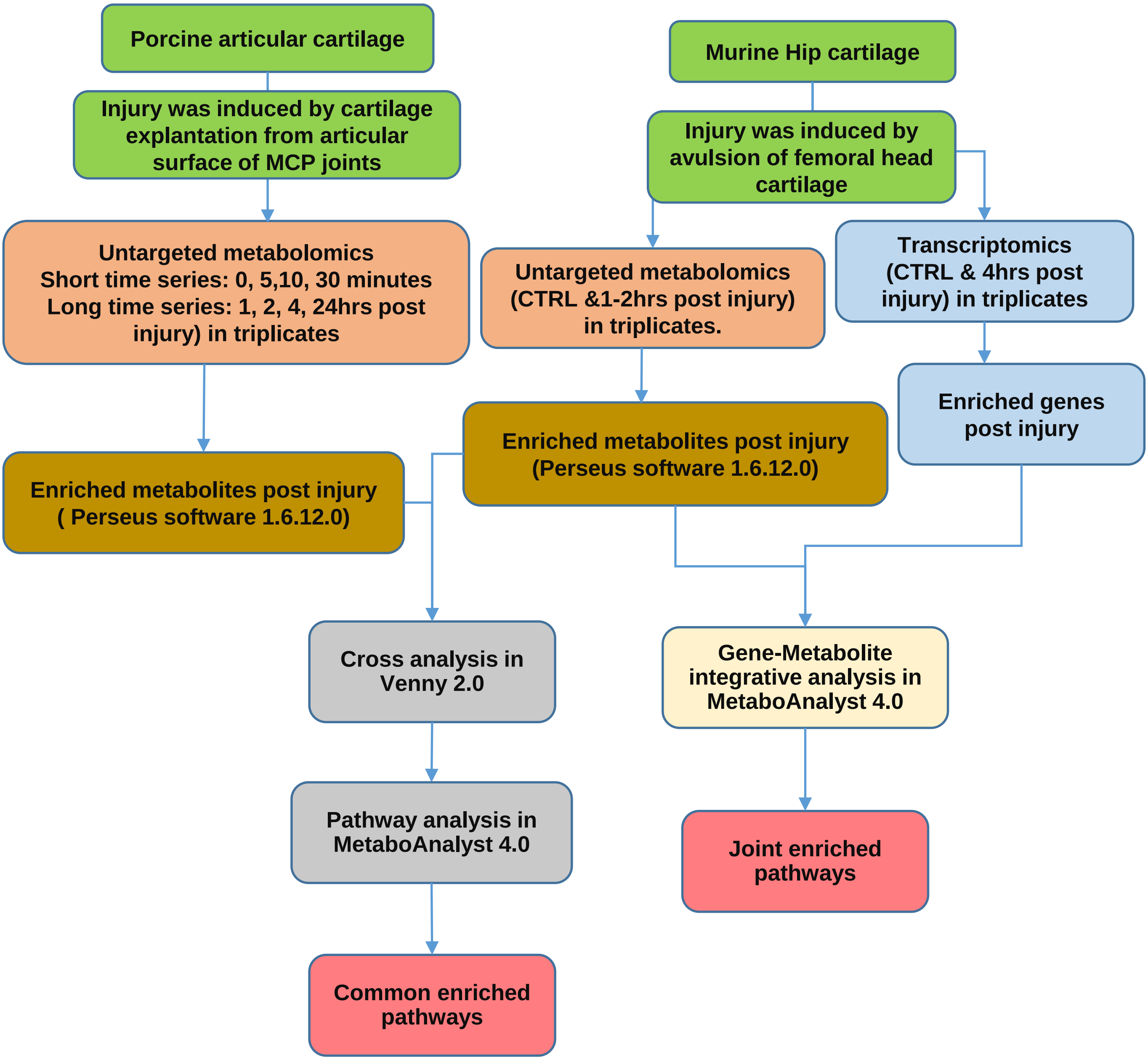

Supplementary Figure 1: Study design and analysis pipeline.

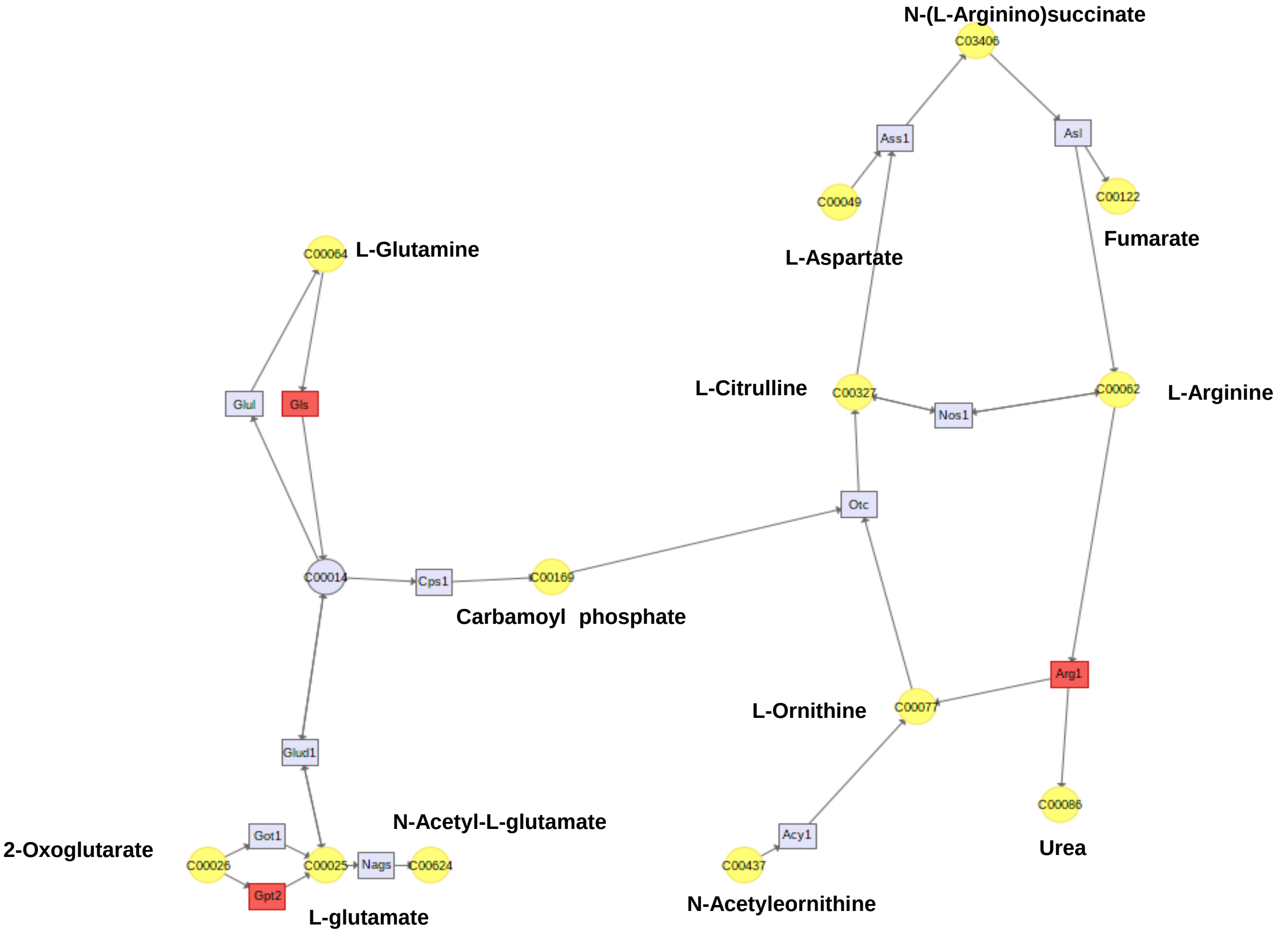

Supplementary Figure 2: Integrative analysis shows genes and metabolites enriched in arginine biosynthesis pathway. Gene list with fold of change were co-analysed with enriched metabolites(KEGG ID) using joint analysis module in MetaboAnalyst 4.0). Enriched metabolites are labelled and colored in yellow. Genes in red are upregulated post injury.

Arginine and proline metabolism

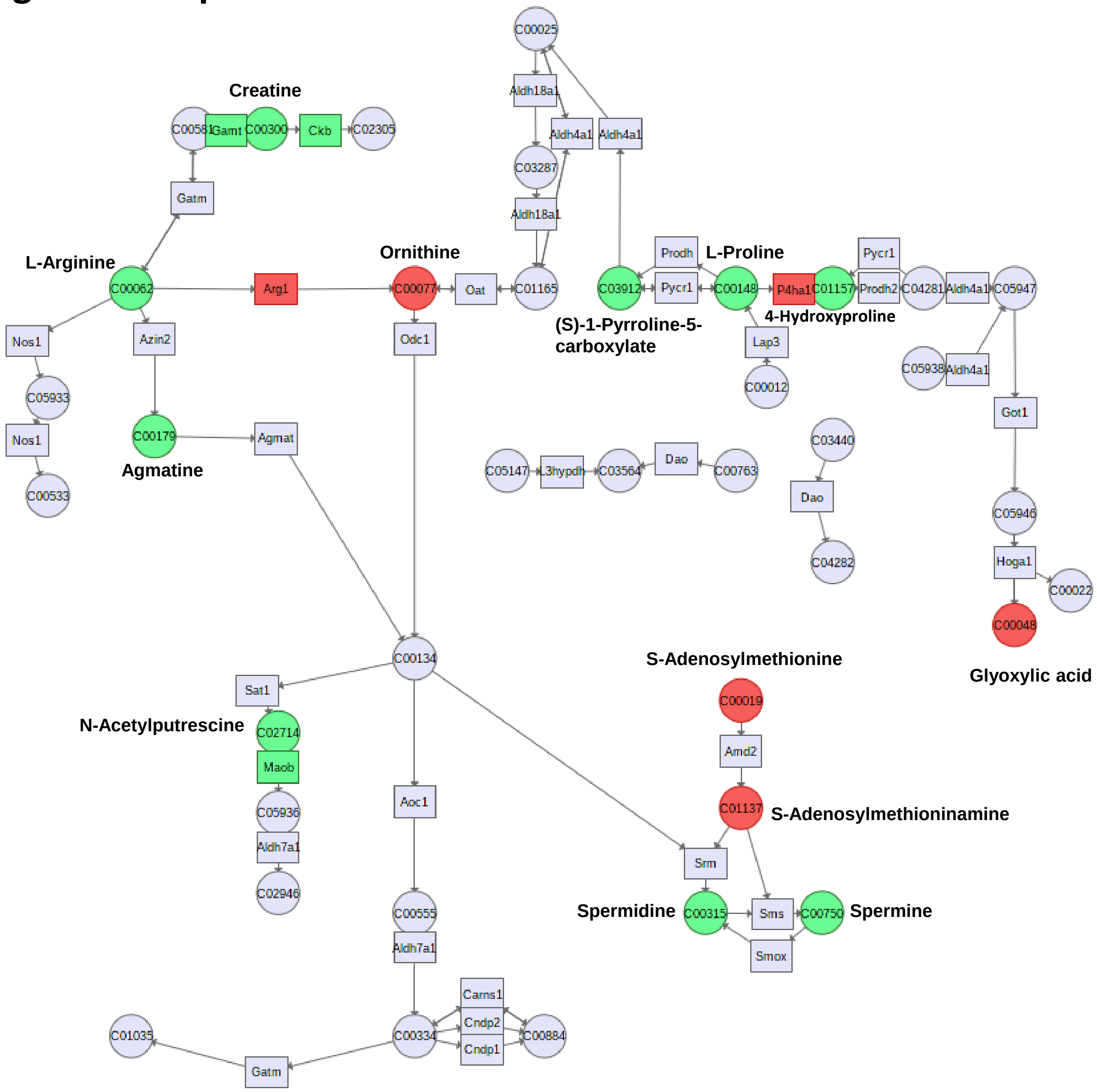

Supplementary Figure 3: Integrative analysis shows genes and metabolites enriched in arginine and proline metabolism . Genes list with fold of change post injury was co-analysed with enriched metabolites(KEGG ID) using joint analysis module in MetaboAnalyst 4.0. Enriched metabolites and genes post injury are shown in red(upregulated) and in green (downregulated).

Glycolysis/Gluconeogenesis

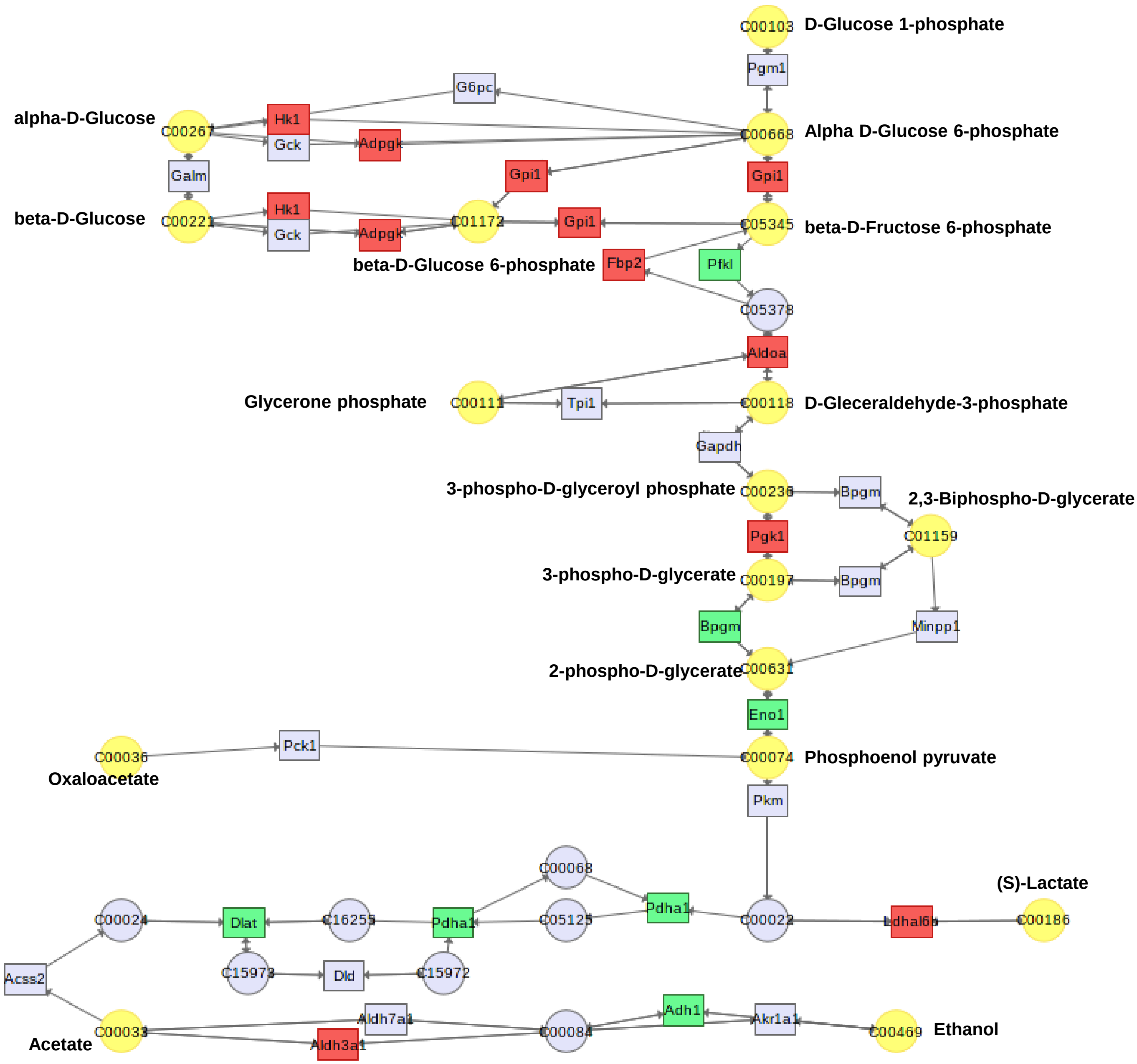

Supplementary Figure 4: Integrative analysis shows genes and metabolites enriched in glycolysis/gluconeogenesis pathway. Genes list with fold of change was co-analysed with enriched metabolites(KEGG ID) using joint analysis module in MetaboAnalyst 4.0). Enriched metabolites are labelled and colored in yellow. Genes in red are upregulated and in green are down regulated post injury.

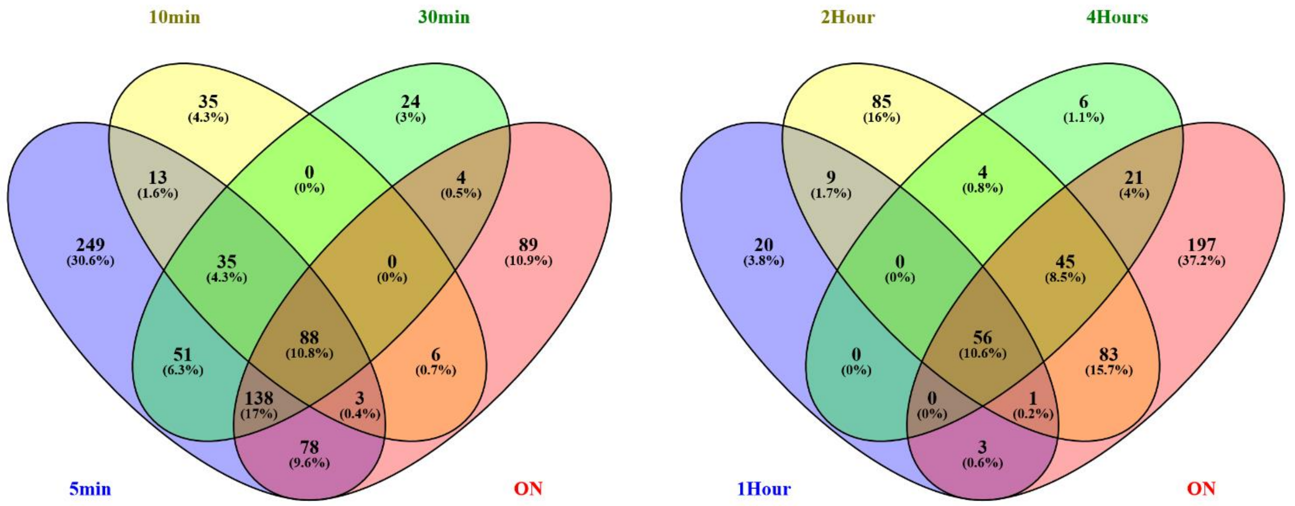

Time points	total	Enriched putative metabolites features
10min 1hr 2hrs 30min 4Hrs 5min ON	48	Vernoflexuoside Dimethamine Abyssinone V 2-phenylacetamide L-homocysteine Khellol glucoside (Z,Z,Z)-3,6,9-Dodecatrien-1-ol Pteryxin N-acetyl-L-glutamate-5-semialdehydeM Peucenidin metabolite Samidin isopentenyladenine Calanolide A 4-hydroxy-L-threonine sebacic acid 4-hydroxyphenylpyruvate D-cathine beta;-D-galactose Sophoraisoflavone A S-adenosyl-L-methionine D-mannose 5,6,7,8-tetrahydropteridine 1-sinapoyl-D-glucose 6,8-Diprenylnarigenin enol-phenylpyruvate Nodakenin spermine p-aminobezoic acid L-sorbose homocysteine 5-aminopentanoate guanine n-octane 5'-Prenylhomoeriodictyol 8-hydroxypurine Alkannin B,B-dimethylacrylate S-methyl-5-thio-D-ribose se-Methyl-L-selenocysteine bleekerine adenine sinapoyl glucose ester NG,NG-dimethyl-L-arginine Cascarillin kynuramine Ginkolide A beta;-D-glucose Isoamidin
ON	48	sulfate (+)-(1R,2R)-1,2-diphenylethane-1,2-diol 5-formyl-tetrahydrofolate Ranunculin N6-(1,2-dicarboxyethyl)-AMP 4-methyl-5-(2-phosphoethyl)-thiazole tryptophan deoxyribose-5-phosphate L-tyrosine dethiobiotin Elemicin 4-diphosphocytidyl-2-C-methylerythritol 2-amino-7,8-dihydro-4-hydroxy-6-(diphosphooxymethyl)pteridine 4-amino-2-methyl-5-phosphomethylpyrimidine 4-hydroxy-4-(3-pyridyl)-butanoate Aethusin Malvalic acid chorismate Sterculic acid cardiospermin adifoline 5,10-methenyltetrahydrofolate Stearolic acid leukotriene-C4 (3'R,$'R)-3'Epoxyangeloyloxy-4'-acetoxy-3',4'-dihydroseselin Rutarin (E,E,E)-N-(""-methylpropyl)hexadeca-2,6,8-trien-10-ynamide Tuliposide A L-tryptophan 6-lactoyl-5,6,7,8-tetrahydropterin L-homoarginine linoleic acid inosine phloretin 5,6,7-Trimethoxycoumarin deoxyribose-1-phosphate sarmentosin formylkynurenine O-succinyl-L-homoserine ribose-1-arsenate Dimethyl disulfide prephenate N,N-dimethyltryptamine cinchonine harmaline oxaloglutarate 5-formiminotetrahydrofolate dihydrobiopterin

Supplementary Figure 5: Cross analysis of metabolites features enriched at different time points post injury to porcine articular cartilage.
